# Simulation of Regional Karst Aquifer System and Assessment of Groundwater Resources in Manatí-Vega Baja, Puerto Rico

**DOI:** 10.4236/jwarp.2015.712075

**Published:** 2015-08-20

**Authors:** Balati Maihemuti, Reza Ghasemizadeh, Xue Yu, Ingrid Padilla, Akram N. Alshawabkeh

**Affiliations:** 1 Department of Civil and Environmental Engineering, Northeastern University, Boston, MA, USA; 2 Department of Civil Engineering and Surveying, University of Puerto Rico, Mayaguez, PR, USA

**Keywords:** Karst Aquifer, Groundwater Modeling, Seawater Intrusion, MODFLOW, Puerto Rico

## Abstract

The North Coast karst aquifer system of Puerto Rico, the most productive aquifer of the island, is a vital water source for drinking water and local ecosystems. High freshwater demands alter the coastal groundwater system that impacts both human populations and coastal ecosystems of the island. To predict how this system might respond to rainfall events and high pumping demands, we used the equivalent porous medium (EPM) technique to develop a three-dimensional ground-water flow model to estimate hydrogeological parameters and assess groundwater resources in the Manatí-Vega Baja karst aquifer. The approach is based on the hypothesis that the simplified EPM approach will reproduce groundwater hydrodynamics in this complex karst environment. The steady-state model was calibrated with trial and error and parameter estimation methods using an observed groundwater table of 1995 (r = 0.86, p < 0.0001, n = 39). The large-scale simulation suggested that groundwater flow roughly follows the elevation slope [*i.e*. south to north). Calibrated hydraulic conductivities range from 0.5 to 86 m/d, whereas the hydro-geologic data strongly suggest higher permeability in the middle karst section of the study area. The transient model adequately estimates the observed groundwater fluctuations in response to rainfall events from 1980 until 2014. The transient results indicate that the conceptual model accuracy is more acceptable with a mean error (ME) of −0.132 m, mean absolute error (MAE) of 0.542 m and root mean square (RMSE) error of 0.365 m. The results of water budget simulation show that the total recharge satisfies the total groundwater withdrawal rate in the past, but continuous closure of more contaminated wells causes groundwater levels to increase in the future. The results indicate that the assumption of applicability of EPM approach is sustained and supported by measured data in the study area. Taking future water demands into account, this model could be applied further to predict the changes of groundwater levels and mass balance under different exploitation scenarios.

## 1. Introduction

Numerical modeling is an important predictive tool for managing groundwater resources. Groundwater models can be used to test or refine different conceptual models, estimate hydraulic parameters, and most importantly for water resource management to predict how the aquifer might respond to changes in pumping demands and climate change [1]. Groundwater flow models are helpful in determining the direction of groundwater flow, distribution of hydraulic heads and flow magnitudes.

Simulating groundwater flow and accurately assessing groundwater resources are more complex in karst aquifers due to higher uncertainty in hydraulic parameters. Karst terrains are underlain by highly soluble rocks (such as limestone or dolomite) with well-developed secondary porosity, exhibiting distinctive hydrology and landforms such as sinkholes, sinking streams, springs, and caves [2]-[4]. A more complete understanding karst formation and hydrogeological characteristics would improve the simulation modeling accuracy [5] [6].

Karst aquifers show very high spatial heterogeneity [7] [8]. The EPM method is one of simplest yet most debated approach to characterize karst aquifer systems [2] [9]-[14]. EPM assumes that a karst aquifer is equivalent to a porous medium in which karst channels and large faults are treated as areas of higher hydraulic conductivity [10] [15]. In karst systems, the concept of a representative elementary volume is used when the size of the area of interest (or the cell in a model) becomes large enough to approximate equivalent porous media [16] [17]. Although accurate simulation of transport processes is still problematic in small (local) scales, one may be able to model hydraulic heads, flow volumetric, and general flow directions by dealing with the complex aquifer as a porous media, especially in regional-scale and intermediate-scale studies [18].

Karst aquifers contribute to a major portion of the groundwater supply in Puerto Rico (PR) as well as in many parts of the world. Karst covers between 28% [19] and 34% of Puerto Rico [20] and is most extensive in the northern karst belt, which is well documented by several USGS reports [20]-[22].

Public-water supply (PWS) withdrawals within the Manatí quadrangle area constitute about 70% of the total water demand within the municipalities of Manatí and Vega Baja. Groundwater conservation and resource distribution in subsurface systems have elicited considerable interest from both public and private sectors. Due to environmental contaminants, the Manatí-Vega Baja area upper aquifer received more concerns recently. Some studies were conducted to estimate groundwater flow, such as Gregory (2001), which estimated its water budget using a steady state two-dimensional model, but their model did not evaluate the temporal trend and dynamics of the aquifer. The objective of this study is to use the EPM approach to establish a three-dimensional groundwater flow model and to evaluate the adequacy of EPM in reproducing the groundwater flow behavior and temporal water table fluctuations. It is questionable whether simplifying the high heterogeneity, which is often impractical to quantify, allow for accurately simulating the complex karst system. The thrust for the current study is the ability to combine limited field data with numerical simulations to generate groundwater flow estimates in higher scale as well as evaluating the spatial and temporal patterns of the groundwater flow and its influence in the environment. Insight obtained in this study will better characterize the behavior of the aquifer system, assess long-term sustainability of its groundwater, and evaluate the hydrogeological properties of the unconfined upper karst aquifer of the Manatí-Vega Baja area within the North Coast Province of Puerto Rico.

## 2. Site Description

### 2.1. Location

**Figure 1.**
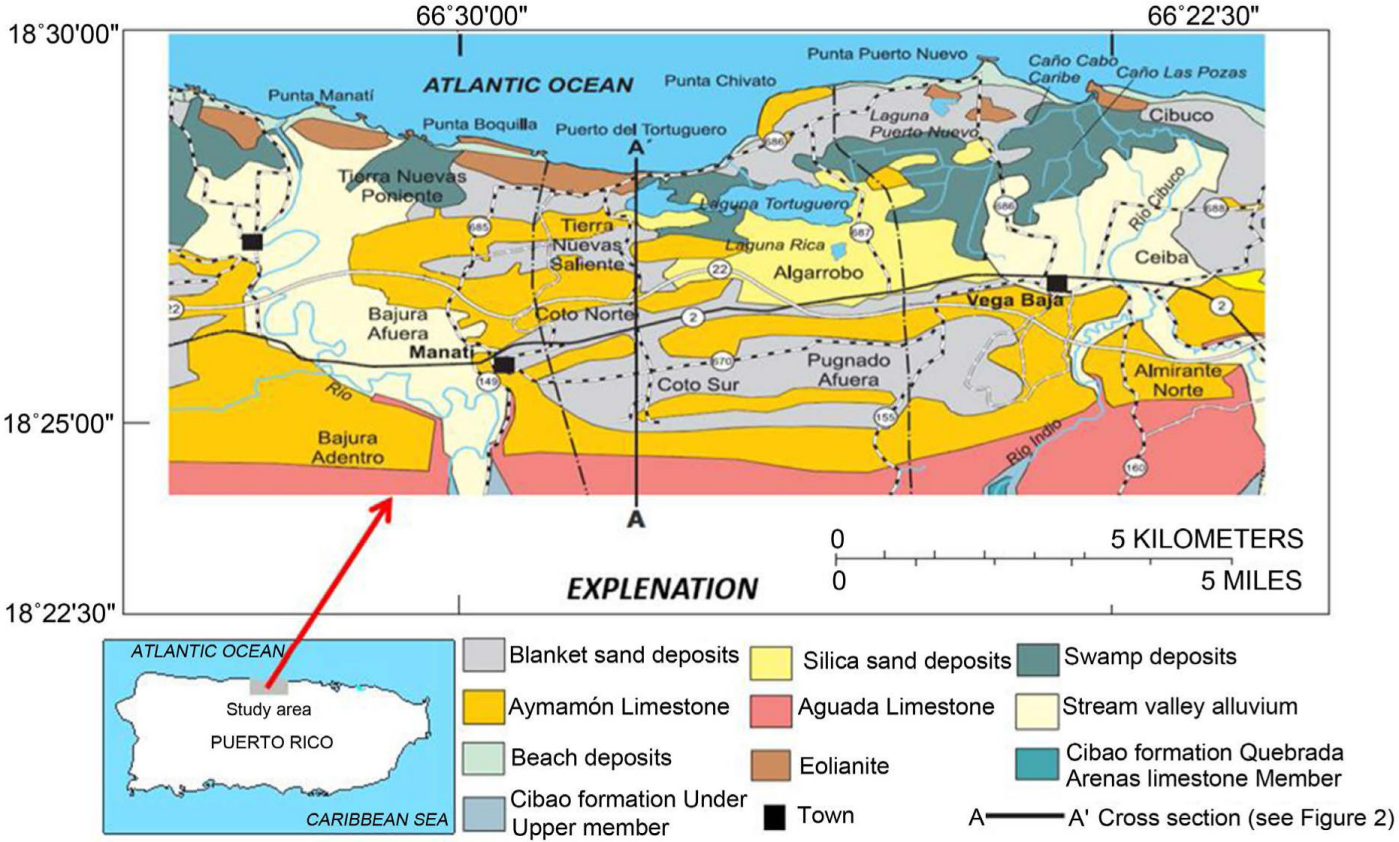
Location of the Manatí-Vega Baja study area and important hydrologic features and generalized surficial geology of the Manatí-Vega Baja study area. (Modified from Monroe, 1980).

The study area of Manatí-Vega Baja is located in north-central Puerto Rico and covers approximately 204.61 km^2^ (Figure 1). This area is bordered by the Atlantic Ocean on the north and by the rugged cone karst topography in the center and to the south. The western boundary is a north-south line, just west of the Río Grande de Manatí, and the eastern boundary is a north-south line on the east of the confluence of the Río Indio and the Río Cibuco.

### 2.2. Geological Setting

Tertiary limestone within the North Coast Province generally strikes to the west and dips to the north with slope of 6 to 7 degrees, near the contact with volcanic rocks south of the study area, and from about 1 to 2 degrees near the coast [23]. These rocks, grouped into six formations, are named in ascending order: the middle Oligocene San Sebastián Formation, the late Oligocene Lares Limestone, the Oligocene and Miocene Cibao Formation, the Miocene Aguada and Aymamón Limestones, and the lower Miocene Camuy Formation. Alluvial deposits occur in the river valleys of the Río Grande de Manatí and the Río Cibuco, with a sequence of “blanket sands” overlying limestone in upland areas (Figure 1).

### 2.3. Hydrology and Hydrogeological Setting

**Figure 2.**
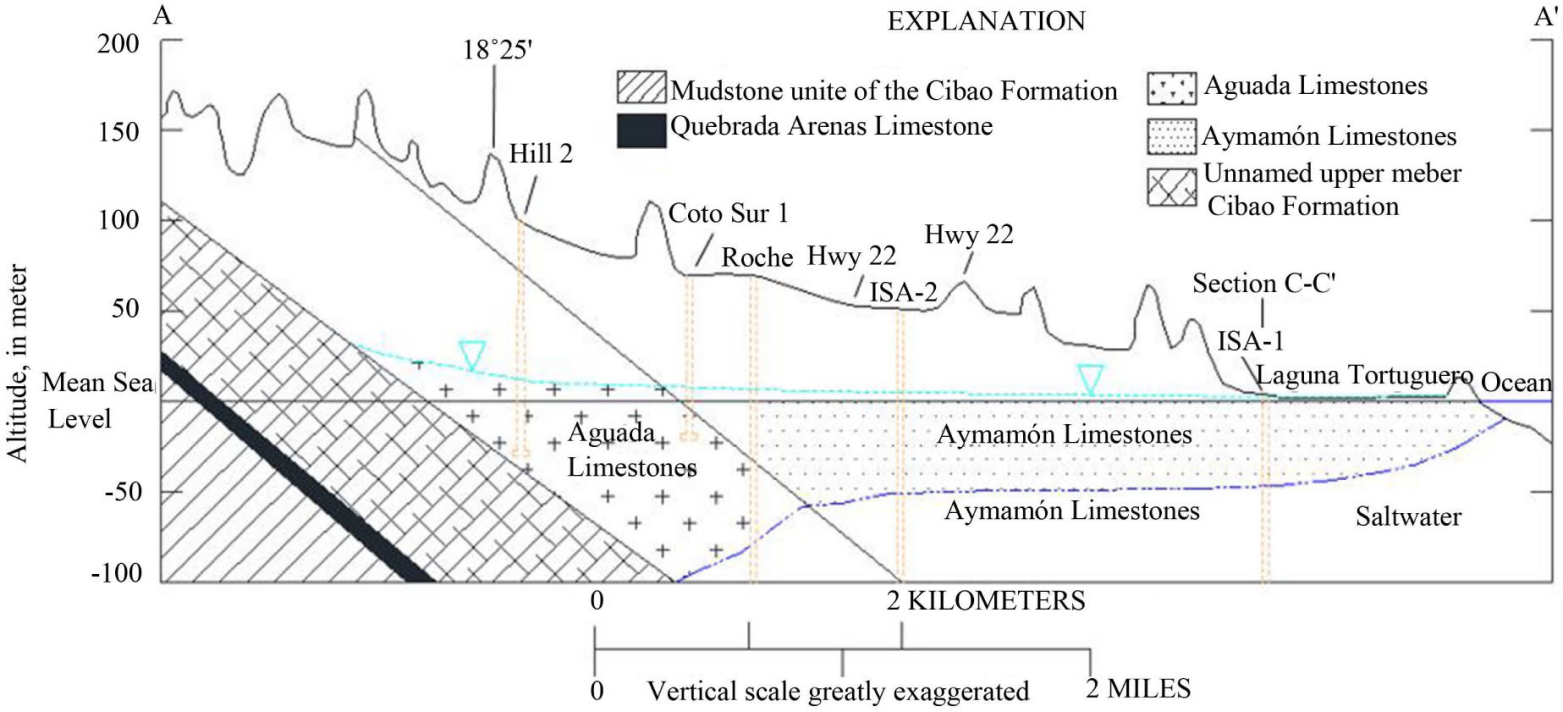
Generalized hydrogeologic cross sections of the Manatí-Vega Baja study area, North Coast Province, Puerto Rico Refer to Figure 1 for cross-section location.

The North Coast karst groundwater system contains two major aquifers. The upper water-table aquifer lies within the Aymamón and Aguada limestone and alluvial deposits along the coastal areas, whereas the lower aquifer occurs within various locations of the Cibao Formation and the Lares limestone (Figure 1 and Figure 2). The upper aquifer is connected to the surface throughout most of its outcrop area. The lower aquifer is confined toward the coastal zone and outcrops to the south of the upper aquifer, where it is recharged. Groundwater in upper aquifers flows from recharge areas toward discharge areas near the coast, streams, wetlands, springs, and other surface-water features [24]. Long-term records from National Weather Service rainfall stations in the study area indicates a relative dry season from December to April, and a wet period from May to November, associated with the hurricane season [25]. The mean monthly rainfall records indicate that May is the wettest month, followed closely by November; while February and March are the driest months. Average annual rainfall in the study area ranges from less than 1.5 m near the coast to 1.8 m in the karst terrain near the southern boundary.

Although some reaches of rivers and streams are important sources of groundwater recharge, most aquifer recharge in this region is from infiltration of rainfall through the limestone outcrops via runoff to sinkholes and enclosed topographic depressions. Within these areas, recharge, which has been estimated by groundwater flow models, is in the range of 0.52 to 0.51 m/yr. The average annual recharge to the upper aquifer of the study area was estimated to be about 0.52 χ 10^8^ m^3^ to 1.04 χ 10^8^ m^3^ [26]-[28].

**Figure 3.**
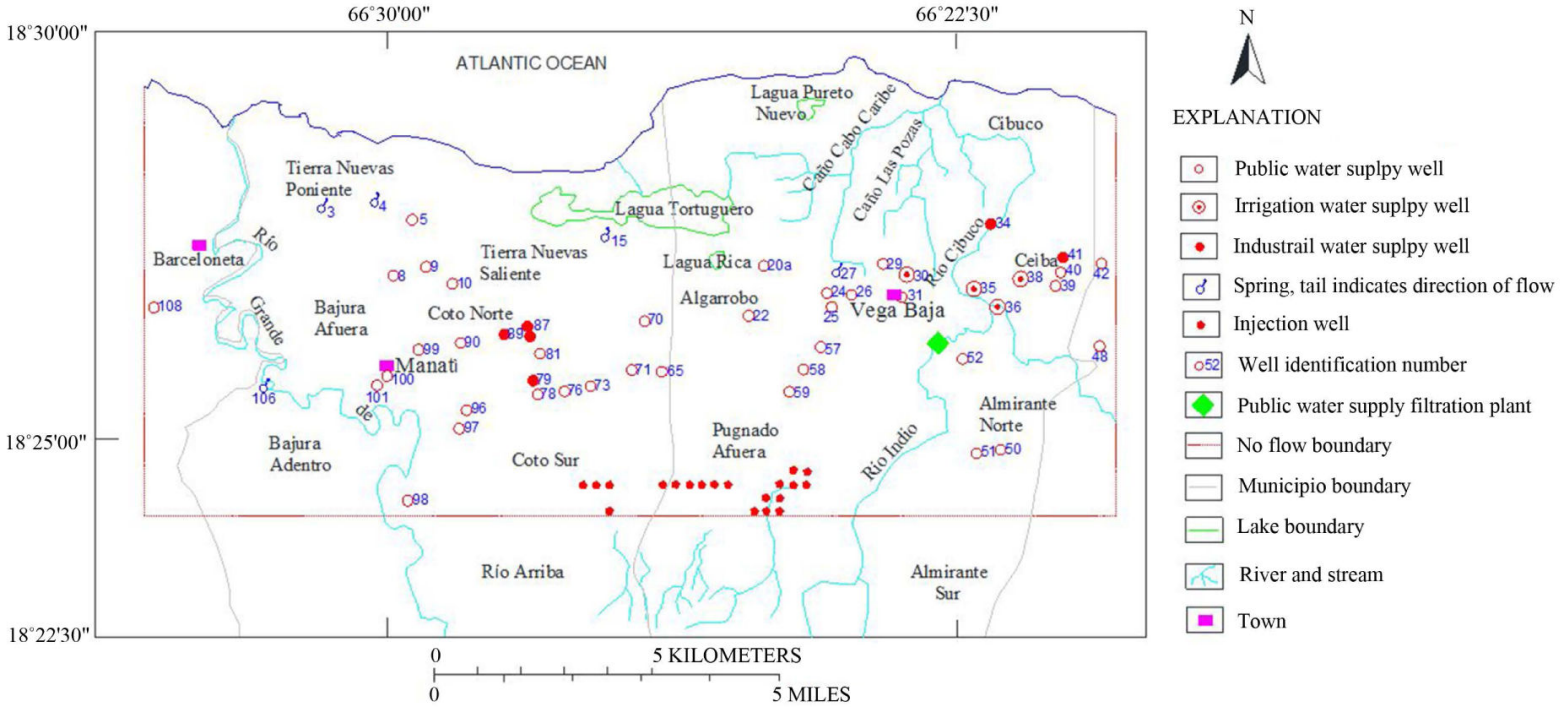
Conceptual model boundary map of the study region.

Prior to the 1940s, the Upper Aquifer System in the Manatí-Vega Baja region was relatively undeveloped. Drilling activities started in the early 1960s and an extensive drilling program accelerated in the 1970s. In 2014, the total number of pumping wells has reached 120, and the total annual groundwater extraction has continually increased. The locations of productive wells used to define the upper aquifers and confining unit are shown in Figure 3. The groundwater withdrawal rates were 7344, 22,974, 55,509 and 65,488 m^3^/d in 1945, 1970, 1980 and 1995, respectively.

The groundwater of the Upper Aquifer System generally occurs under unconfined conditions in the Manatí-Vega Baja area. The water table is not stable and fluctuates during wet and dry seasons and also influenced by the withdrawal rates. Four observation wells (Table 1) are used to monitor the water level fluctuations of the Upper Aquifer. These wells have total depths above mean sea level (MSL) of 38.41 m, 34.75 m, 83.82 m and 76.2 m, respectively.

Besides groundwater withdrawals, the next prominent groundwater discharge feature for the upper aquifer is Laguna Tortuguero. The mean annual groundwater discharge into Laguna Tortuguero was estimated as 0.6 m^3^/s by Bennett and Giusti [29], which was comparable to the 0.62 m^3^/s mean annual discharge rate through the ocean outlet channel obtained by Quiñones and Fusté [30].

## 3. Methodology

To simplify the complex field system and organize available data, an approximated representation of field situation is reconstructed through MODLFOW [31] implemented in an advanced graphical preprocessing and post processing program, Groundwater Modeling System (GMS v.10, Aquaveo 2014). While simplified under Equivalent Porous Media (EPM) approach, the developed conceptual model retains enough complexity in representing hydrological conditions to sufficiently reproduce the groundwater behavior. The conceptual model for Manatí-Vega Baja region adapted by this study consists of three-dimensional hydrogeological layers, representing different layers of the unconfined upper limestone aquifers. Hydrogeological maps and data were imported into GMS coverage layers and were used as input for developing the conceptual model. Coverages were also used to define flow budgets, model boundary condition, sources and sinks, observation wells, elevations, and recharge and hydraulic conductivities. The conceptual model was then converted into a 3D numerical MODFLOW Framework, where the mathematical calculations are performed, and to reproduce the groundwater heads, water flow budget, and water flow velocity and flow directions.

### 3.1. Model Setup

The model domain, which was divided into 100 columns and 70 rows, included 6789 grid cells. The western and eastern edges of the model area are no-flow boundaries (Figure 3). These boundaries were extended beyond the rivers to simulate the surface-water/groundwater interaction, and to minimize computational errors resulting from the effects of model boundaries. The southern surface runoff entering the aquifer was represented in the model by injection wells located in south of the model domain. The bottom altitude of the aquifer is a no-flow boundary condition at depth of −100 m above MSL. Figure 3 shows the location of productive wells, springs, filtration plants and lagoons as well as river and stream direction and longitudes in the Manatí-Vega Baja study area. North of the blue line, the lower boundary of the upper aquifer is delimited by the saltwater/freshwater interface. The southern, western and eastern edges of the model area are no-flow boundary.

The River Package was used to simulate the interaction between the upper aquifer and Laguna Tortuguero (Figure 2). The seabed along the coast of the Atlantic Ocean was represented as a streambed with a head of 0 m. The major springs in the study area were simulated using the Drain Package within MODFLOW [31]. Using the mass-balance approach, a maximum aquifer recharge rate for the karst terrain in northern Puerto Rico was established at 0.51 to 0.64 m/yr [32]. Initially for the steady-state model, the recharge rate for areas with closed depressions or areas with poorly defined surface-water drainage was assigned a maximum value of 0.46 m/yr.

### 3.2. Steady State Calibration

To establish that the model can reproduce measured field data, MODLFOW was used together with Parameter Estimation [33] implemented in GMS. Calibrating groundwater flow and accurately adjusting the hydrogeologic parameter values are more complex in karst aquifers due to high uncertainty in hydraulic parameters. Model calibration is the essential to anticipate and predict the geologic uncertainties, where model inputs are modified until the resultant predictions give a reasonably good fit to the observed data. In this study, the model was calibrated in steady-state with a trial and error approach using parameter estimation methods matched to the observed groundwater table of 1995 for which sufficient spatially distributed data are available.

Steady state calibration for the flow model was achieved by comparing the hydraulic heads obtained from available groundwater level contour maps or observation wells (n = 39) of the aquifer and the calculated hydraulic heads of the MODFLOW simulation in order to simulate the flow lines of this aquifer layers [34]. Calibration of the steady state model was performed using the root mean square error (RMSE), mean absolute error (MAE), mean error (ME) and mass balance discrepancy of water into and out of the system. The overall RMSE of the steady state model is 1.86 m, MAE is 1.254 m and the ME is 0.212 m for the calibrated wells (n = 39).

**Figure 4.**
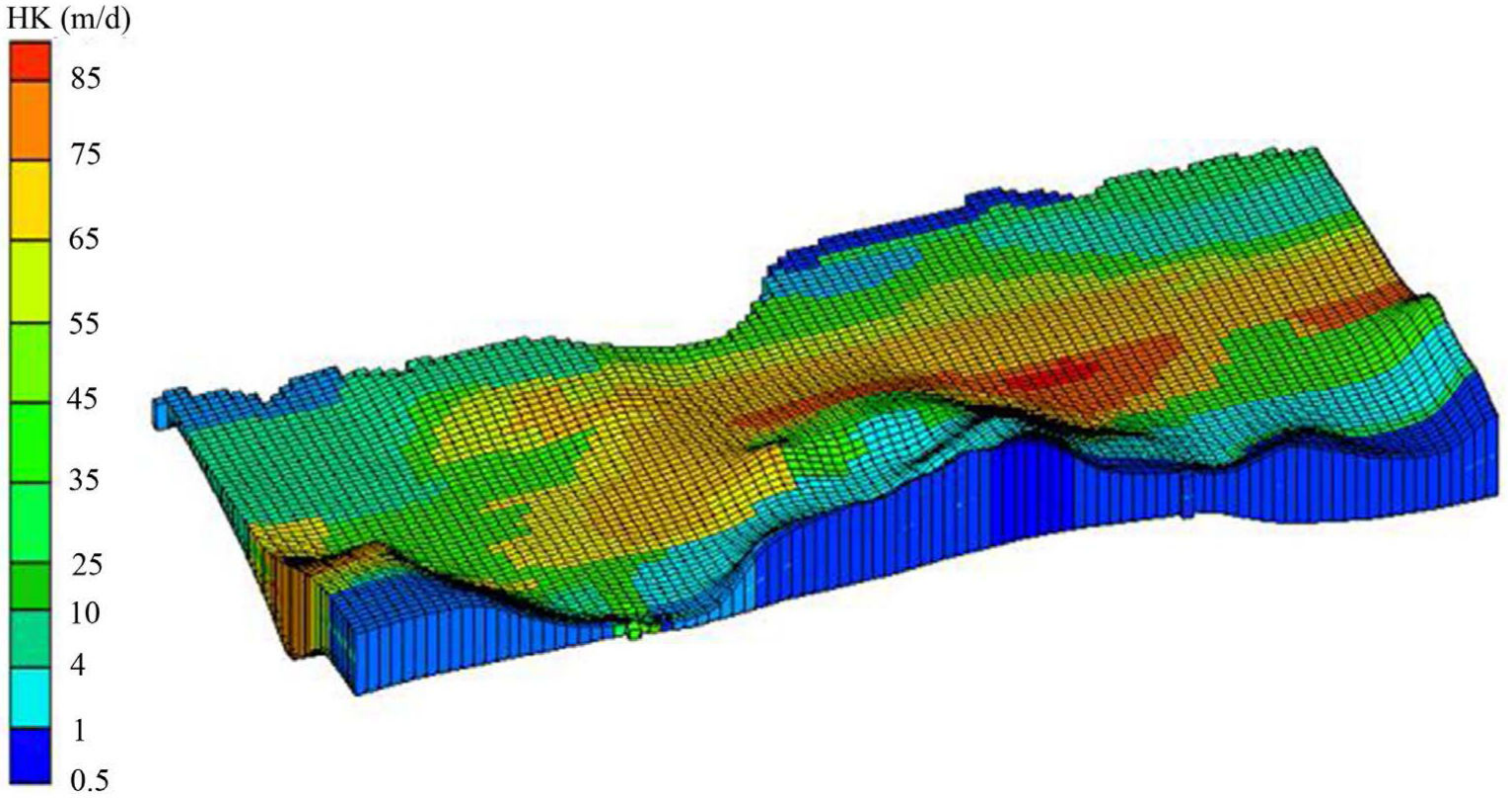
Regional distribution map of hydraulic conductivity.

**Figure 5.**
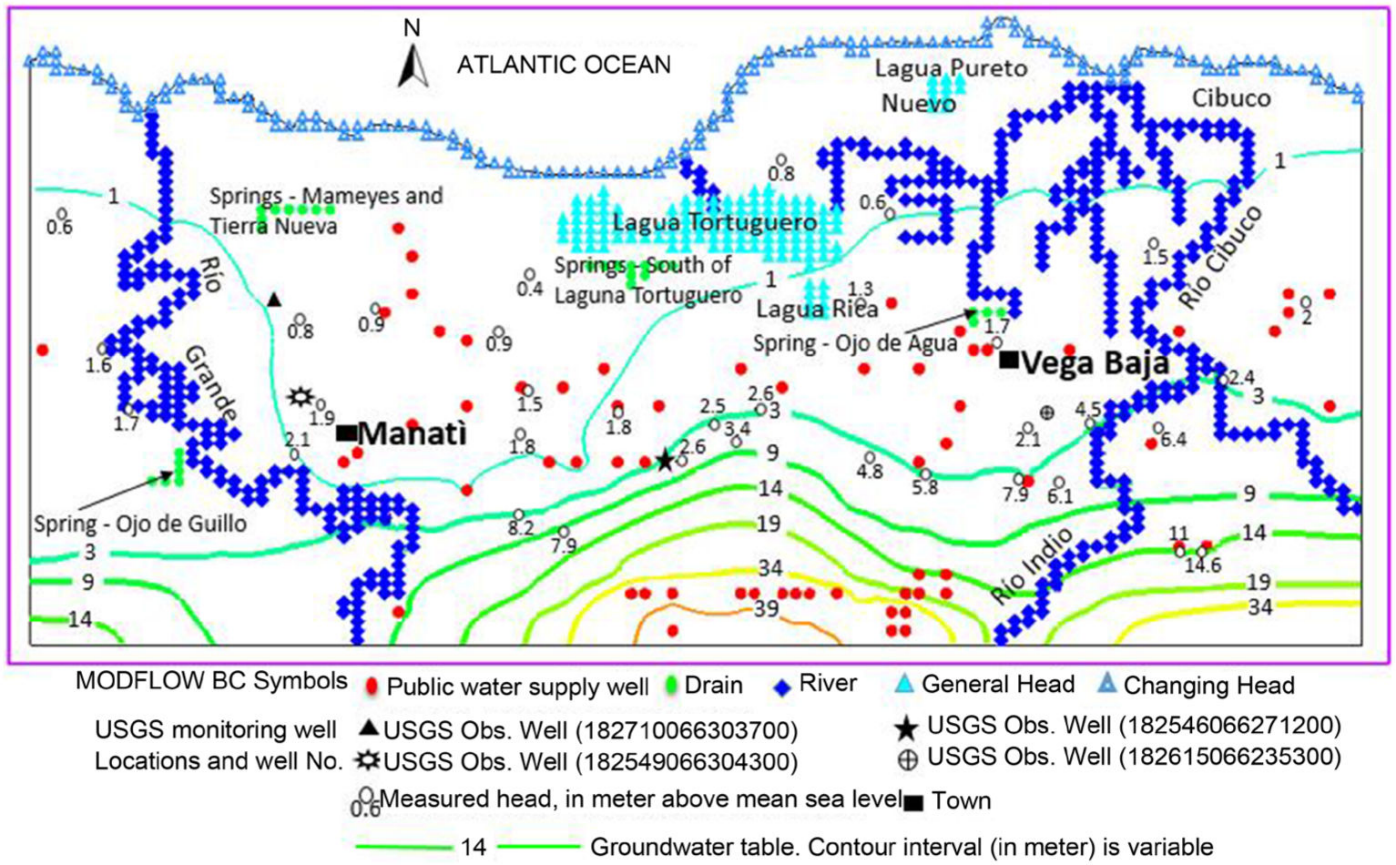
Comparison value of the computed head and observed head.

Horizontal hydraulic conductivity and recharge were initially assigned based on a previous study [35] and then the PEST method was applied to estimate the optimum values for recharge and conductivity within defined specific ranges for each zone. After calibration, horizontal hydraulic conductivity values obtained were between 0.5 m/day to 86 m/day for different units, e.g. limestone, silt, sand, and gravel. In Figure 4, the red color area represents the higher transmissivity zones, which appears in the center of the study area, near Manatí, and southwest of Vega Baja. In these areas, the hydraulic conductivity values decrease both to the north and south within the limestone units, and in the part of the aquifer contained within the alluvial deposits. The water table comparisons between observed versus simulated data are shown in Figure 5. To provide information on the overall match of all the monitoring wells (n = 39).

### 3.3. Transient Calibration

Upon achieving a satisfactory steady state condition, the transient groundwater model was implemented for the 34 year period between 1980 and 2014. The transient model consists of 412 monthly stress periods. Each stress period had 10 time steps and used days as a time unit. The first stress period represented the steady state period with no significant drawdown in the water level. Sufficient pumping data were available for verification from 1980 to 1995 and the model is capable of predicting behavior from 1996 to 2014. The groundwater withdrawal rates used for the transient model simulation correspond to the historical pumping rates reconstructed from PRASA public water-supply and the PRDNER records, and the United States Geological Survey (USGS) water-use database, which includes production data for industrial, public water-supply, and irrigation uses.

The data from 1980 to the end of 2014 were used for transient calibration and from 1990 to 1995 for verification. The initial transient simulation used a storage coefficient of 1 × 10 ^−5^ − 7 χ 10 ^−6^ and specific yield of 0.01 - 0.2 for different zones. The first step of the calibration assigned an initial value for storage coefficients and specific yield for model layer. Calibration was performed using trial and error procedure by changing the specific yield, storage coefficient, and with very limited range in the hydraulic conductivity values. In this study area there were only four USGS monitoring wells with continues measurements. The available data were used to calibrate the transient state of the model. The main limitations of equivalent porous media approach in karst aquifers are failing to capture groundwater hydrodynamics on a local scale, and failing to simulate transient turbulent flow through conduit network and its interaction with the karst aquifer rock matrix. For this reason, the head error range (−1 < h < 1) has been defined for this transient model calibrations.

### 3.4. Sensitivity Analysis

As with any model, there is uncertainty in the measurements of head (datum errors), hydraulic conductivity, estimation of recharge, and well withdrawal rates. It is therefore important to estimate the effect of improperly simulating a specific parameter. The hydrologic parameters that have the greatest impact on simulation results are identified through sensitivity analyses. Also the non-uniqueness of the calibrated model can be evaluated using sensitivity analysis. A sensitivity analysis was conducted to show that the values of the aquifer’s hydraulic properties as estimated by the model calibration procedure were not random and do in fact influence the final results. In other words, significant variations of the simulated value will drive proportional changes of the model results. This sensitivity analysis quantified the dependency of the model to calibrated aquifer parameters, and hydrogeologic stresses such as withdrawals. The effects of the main parameters governing the regional hydrodynamics (*i.e.* recharge, conductivity, and pumpage) on average water levels were evaluated separately by running the model under reduced or enhanced parameter values. The water levels are more sensible to a decrease in conductivity values than to a decrease in pumping conditions, but less sensitive to an increase in conductivity than an increase in pumping conditions. The sensitivity line for pumping is almost linear and therefore the aquifer is moderately vulnerable to over withdrawal activities. As the Figure 9 indicates, the aquifer is the most sensitive to changes in recharge conditions among other parameters, especially a reduction in average recharge significantly drop the average water levels.

## 4. Results and Discussion

### 4.1. Model Calibrations

**Figure 6.**
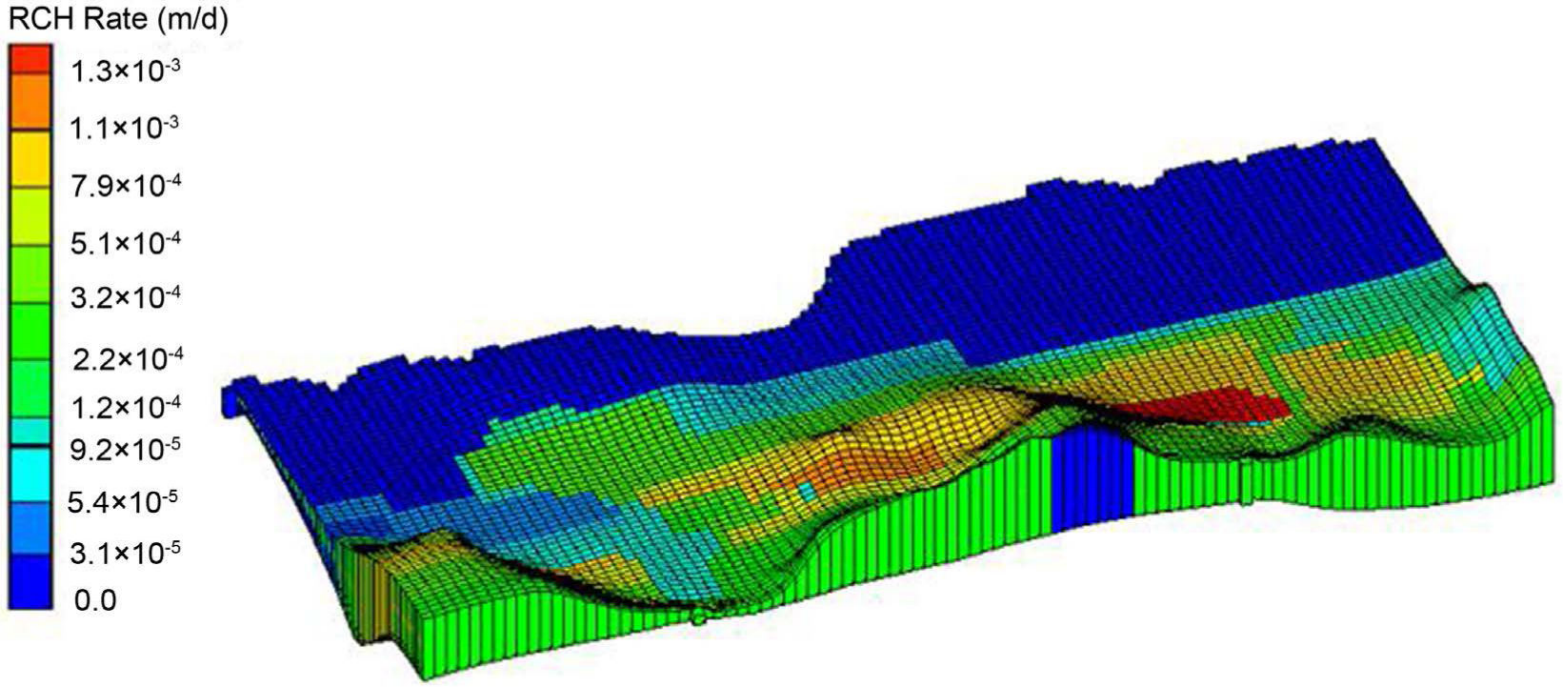
Regional distribution map of recharge rate.

**Figure 7.**
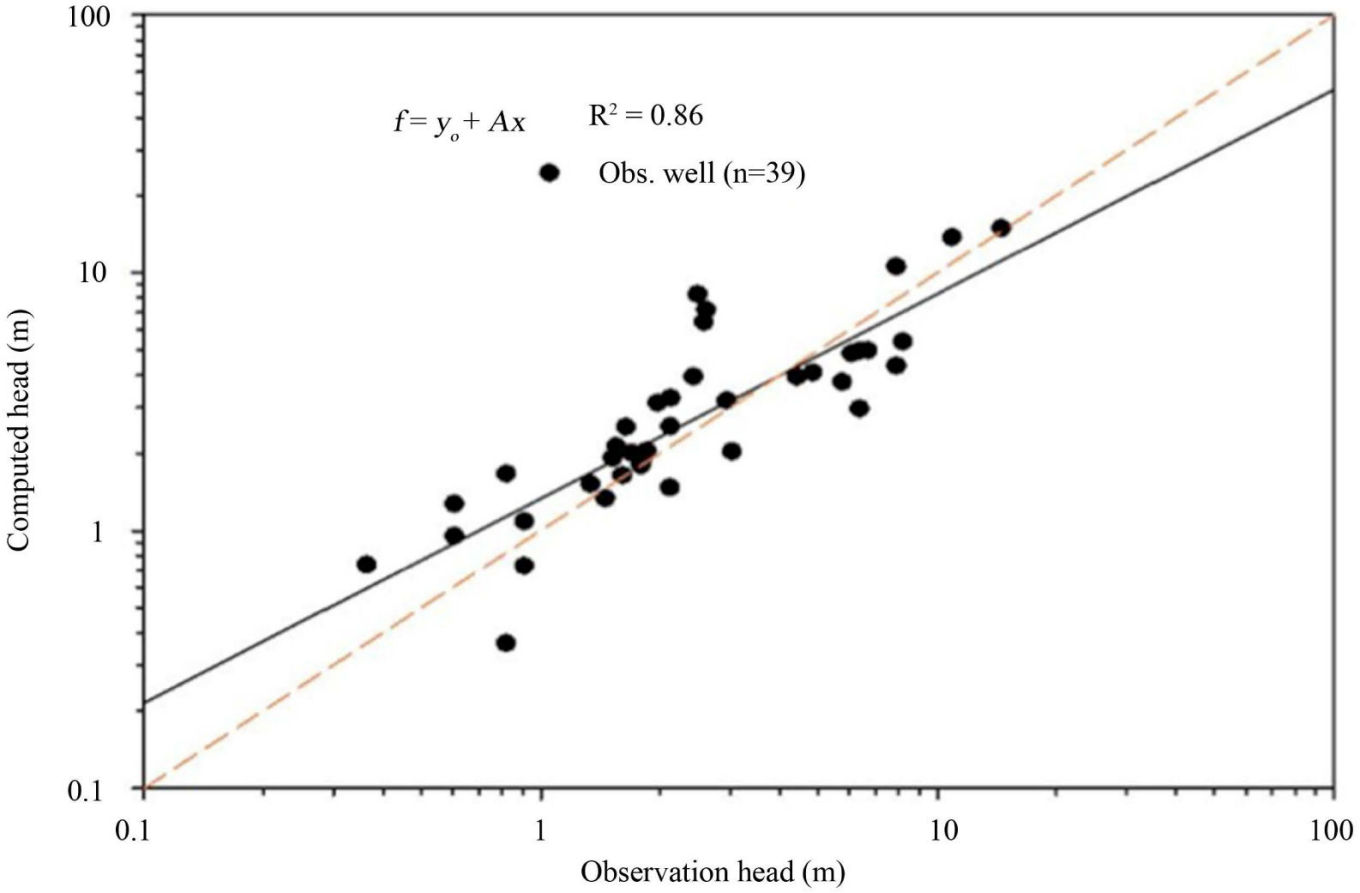
Comparison result of observation and calculation head.

The hydraulic conductivities calibrated through the steady state model ranged from 0.5 to 86 m/d, whereas the hydrogeologic data strongly suggested higher permeability in the middle karsts of the study area (see Figure 4). Simulated aquifer recharge values ranged from 0 m/d in the wetland areas of Cabo Caribe, to 0.0003 m/d in the alluvial valleys of Río Grande de Manatí and Río Cibuco, and 0.0013 m/d in sinkholes and areas of enclosed depressions (Figure 6). Recharge is from infiltration of rainfall through the limestone outcrops via runoff to sinkholes and enclosed topographic depressions. While the high value of recharge appears in the center of the study region, along the north coast (wetland area) the recharge rate is insignificant. The overall RMSE of the steady state model is 1.86 m, MAE is 1.254 m and the ME is 0.212 m for the calibrated wells. The simulated steady-heads are in good agreement with the observed values (r = 0.86, p < 0.0001, n = 39) shown on Figure 7.

The model’s tendency to overestimate or underestimate the actual heads is attributed to the effect of possible conduit or fracture network in this karst aquifer. While pinpointing and simulating the exact location and behaviors of such highly permeable pathways are expensive and in most cases not practical, the employed equivalent porous media approach in our developed model is the easiest (but not most accurate) method available.

The steady state model results show that the computed values are in well-fit to the observed data (Figure 7), indicating that the conceptual model is reasonably calibrated and could be further applied to predict changes in groundwater levels and mass balance under different exploitation scenarios when considering the future water demands in the study area.

**Figure 8.**
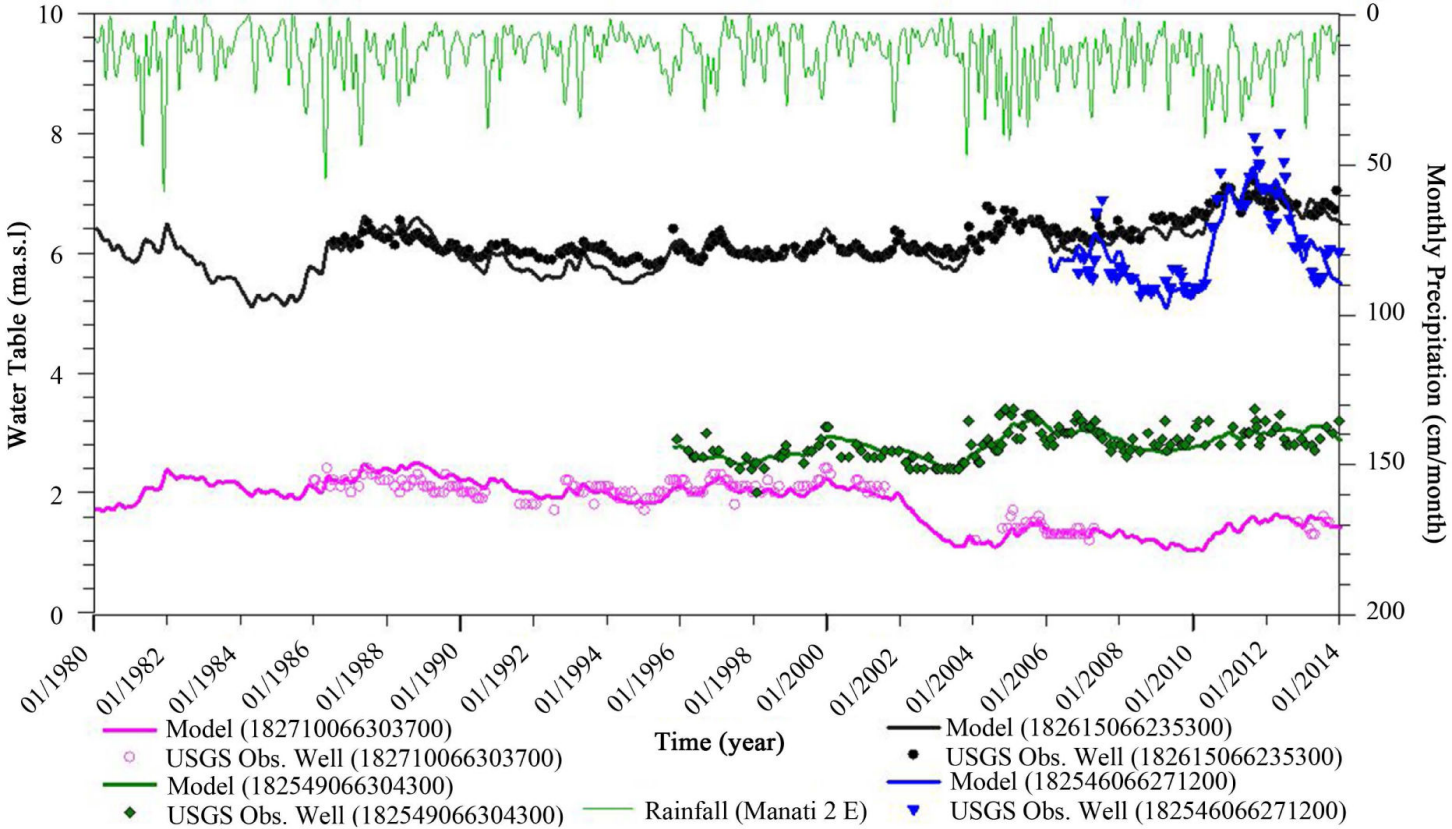
Monthly simulated and observed WT vs. monthly rainfall for 1980-2014. The USGS monitoring wells location showed in Figure 5.

After steady state model calibrations achieved a satisfactory steady state, transient groundwater model was developed for a period of 34 years from 1980 to 2014. The transient calibration results were evaluated by comparing the temporal variations in simulated heads with those of observed water levels at four target observation wells. Figure 8 shows the monthly transient simulation comparison between computed and observed head value. The measured and simulated fluctuations were similar and fit each other well; the head error value is under the target value (−1 < h < 1 m) as shown Figure 8. The transient state calibration results are shown in Figure 8. The overall RMSE of the transient model was 0.365 m, MAE was 0.542 m, and ME was −0.132 m, which all well below calibration target value.

The observed water level data fluctuations matched well with the simulated water level changes resulting from rainfall. For example, groundwater levels in 2011 in the USGS observation (well: 182546066271200) well increased up to a maximum of 2 m according to both measured and simulated data (Figure 8). The fluctuations of the water table in all wells are a function of local groundwater withdrawal trends and the precipitation intensity. As shown in Figure 8, the general magnitude and the sharp fluctuations in water level data (as well as in simulations) are in direct response to monthly rainfall and could be high pumpage in the dry season. Our modeling analyses suggested that aquifer replenishment correlated remarkably with rainfall events both in terms of magnitude and changes.

Figure 8 represents the long term variation of the water table at four USGS wells and the rise in the water table of about 0.4 m at USGS observation well (182615066235300) over the last three decades. The rise is due to the closure of some of the pumping wells over time. Intermediate-term variation in water table corresponds to wet and dry years, as well as groundwater withdrawal activities.

### 4.2. Sensitivity Analysis

**Figure 9.**
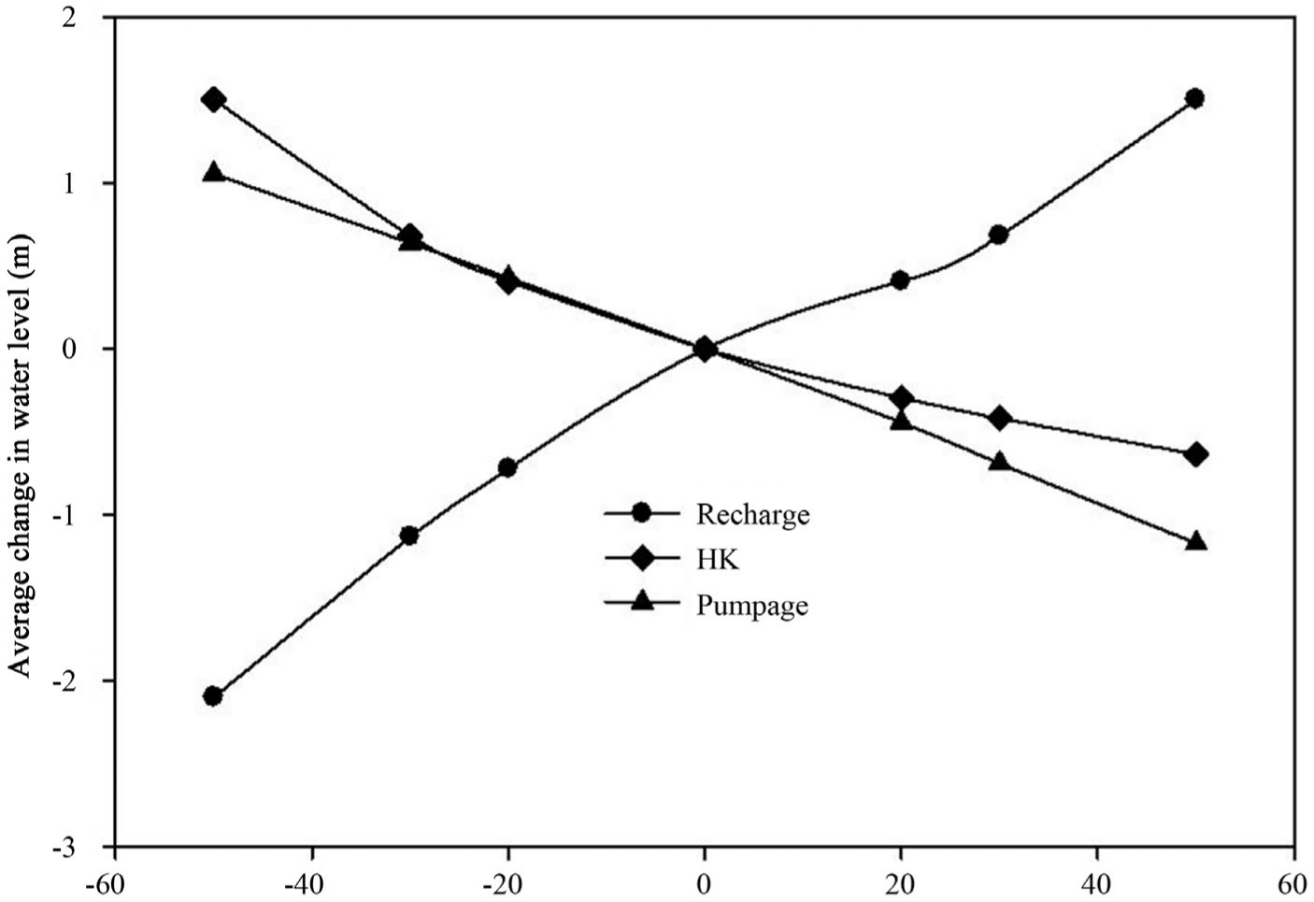
Sensitivity of the predicted water levels to chpanges in recharge, hydraulic conductivity and pumpage rates.

As presented in section 3.4., this karst aquifer is extremely vulnerable to pumping activities as evident by the linear correlation between pumping intensity and the average water levels (Figure 9). A 50% increase in pumping rate would significantly lower the average water level by one meter, and would alter the regional groundwater hydrodynamics. The effect of such over-withdrawal may only be neutralized by about a 30% increase in precipitation. The coastal karst aquifer is especially vulnerable during dry seasons; since a 20% reduction in precipitation would result in lowering the water table by one meter on average. As expected and similar to other karst aquifers, the sensitivity of the water table to hydraulic conductivity is non-linear [15] [36].

The hydrologic parameters that have the greatest impact on simulation results can also be identified through sensitivity analyses. Water levels were most sensitive to variations in recharge and hydraulic pumpage rates (see Figure 9). Simulated water levels increased with increasing recharge and with decreasing hydraulic conductivity.

### 4.3. Water Budget

The water inputs in this system derive from recharge, the constant head (ocean), general head (lake), and river or stream boundaries. The main outputs of water from the aquifer were drainages, river or stream segment and the Atlantic Ocean. The water budget of the entire aquifer obtained from the groundwater flow model is presented in Table 2. The balance between inflows and outflows was consistent with the steady state modeling hypothesis. The flow budget result indicates that the main recharge comes from precipitation, which represents about 76%, followed by 17.9% river, 5.1% sinkholes streamflow, 0.82% lakes and 0.11% Atlantic Ocean. The discrepancy between inflow and outflow was estimated to be 0.04% (shown Table 2), which is a significant improvement of the previous modeling study of the same area (24.27%; [35]).

**Figure 10:**
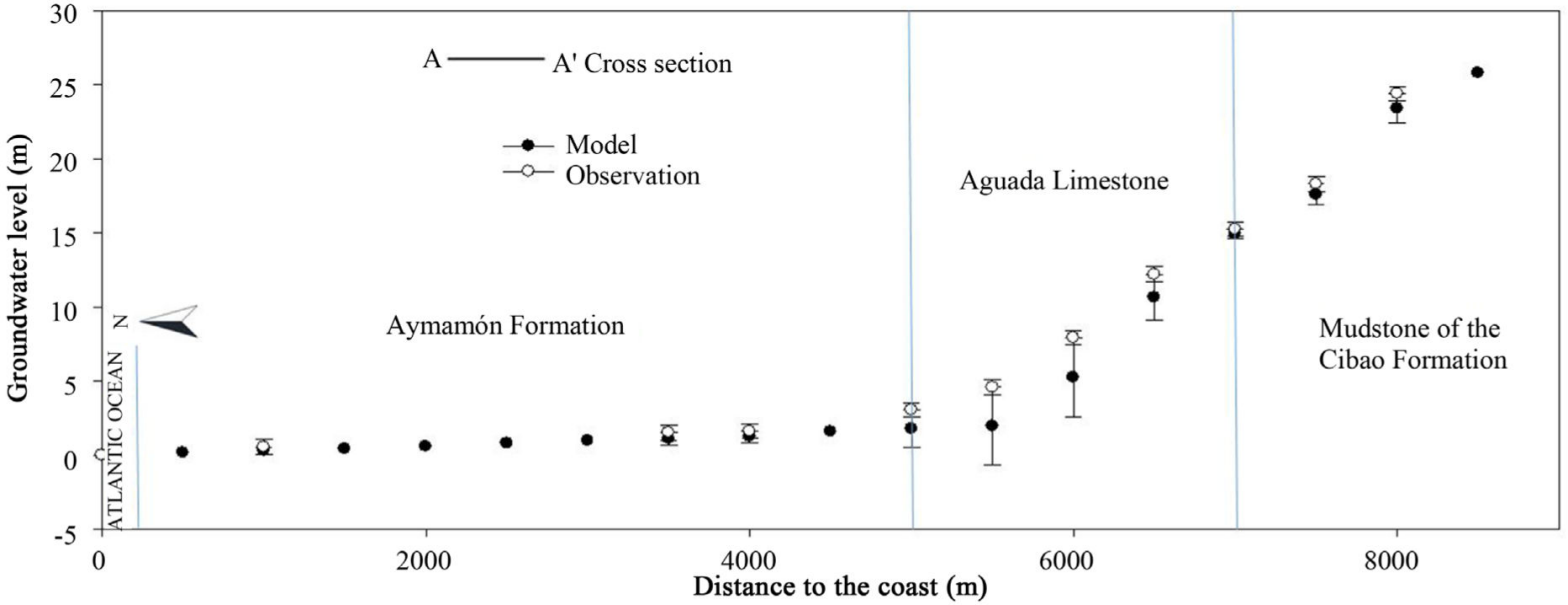
Comparison of simulated and observed groundwater level on cross-section A-A'.

The groundwater levels increased along the cross section A-A', which is from north to south (From Atlantic Ocean to Land; Figure 10). The groundwater flow over the Cibao formation is faster and the flow is thinner due to significantly higher hydraulic gradients. This is typical of all coastal aquifers, and the longitudinal head profiles for springs also follow the same trend with higher heads farther away from the oceans. Farther from the coast (>9000 m), the Aguada formation at the bottom of the aquifer becomes shallower and thinner. Therefore, the storability and conductivity values decrease and the average groundwater level rises with higher rates for areas farther away from the ocean. We also noticed that the magnitudes of seasonal fluctuations of groundwater levels are higher on Aguada limestone. The highest hydraulic gradient occurred in areas close to the southern boundary, which coincident with the lowest values of hydraulic conductivity in this karst aquifer [15]. The water-table gradient is almost flat (25 to 40 meters per kilometer) in the Aymamon limestone. The water table increases to the south in the Aguada limestone (500 to 600 meters per kilometer) toward the model boundary (Figure 10). This hydro diagram demonstrated the groundwater heads in the aquifer of study and its region rising from north to south (from Atlantic Ocean to land).

In the dry seasons, when higher pumping periods decrease the groundwater level, the surface water (river, Atlantic Ocean, stream and lack water) infiltrates the ground and recharges to groundwater. In contrast, the outflow value increases during dry periods. Total ground water pumping ranged from 87,871 m^3^/d to 58,495 m^3^/d. The transient model results indicated that the groundwater level of the aquifer tended to rise in future simulated stress periods.

### 4.4. Flow Field and Seawater Intrusion

**Figure 11.**
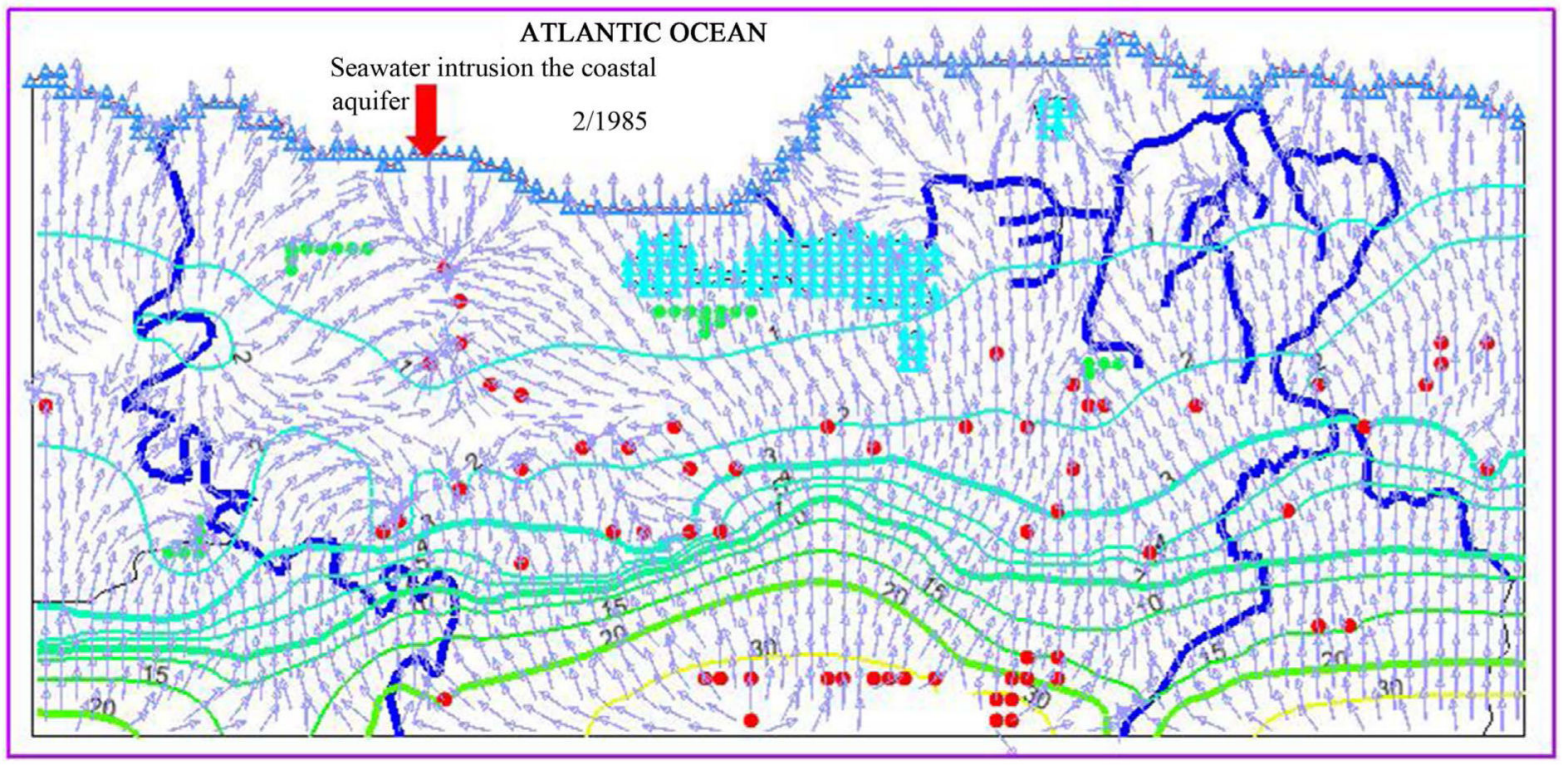
Groundwater level trends, flow directions and seawater intrusion.

In the coastal region of the study area, the model results show that the seawater and groundwater complement each other (Figure 11). Groundwater levels throughout most of the region have been relatively stable or have shown an increasing trend in this study region. In the coastal regions, however, this stability may be misleading as there may be large seawater intrusion since groundwater levels are below sea level throughout much of this region.

**Figure 12.**
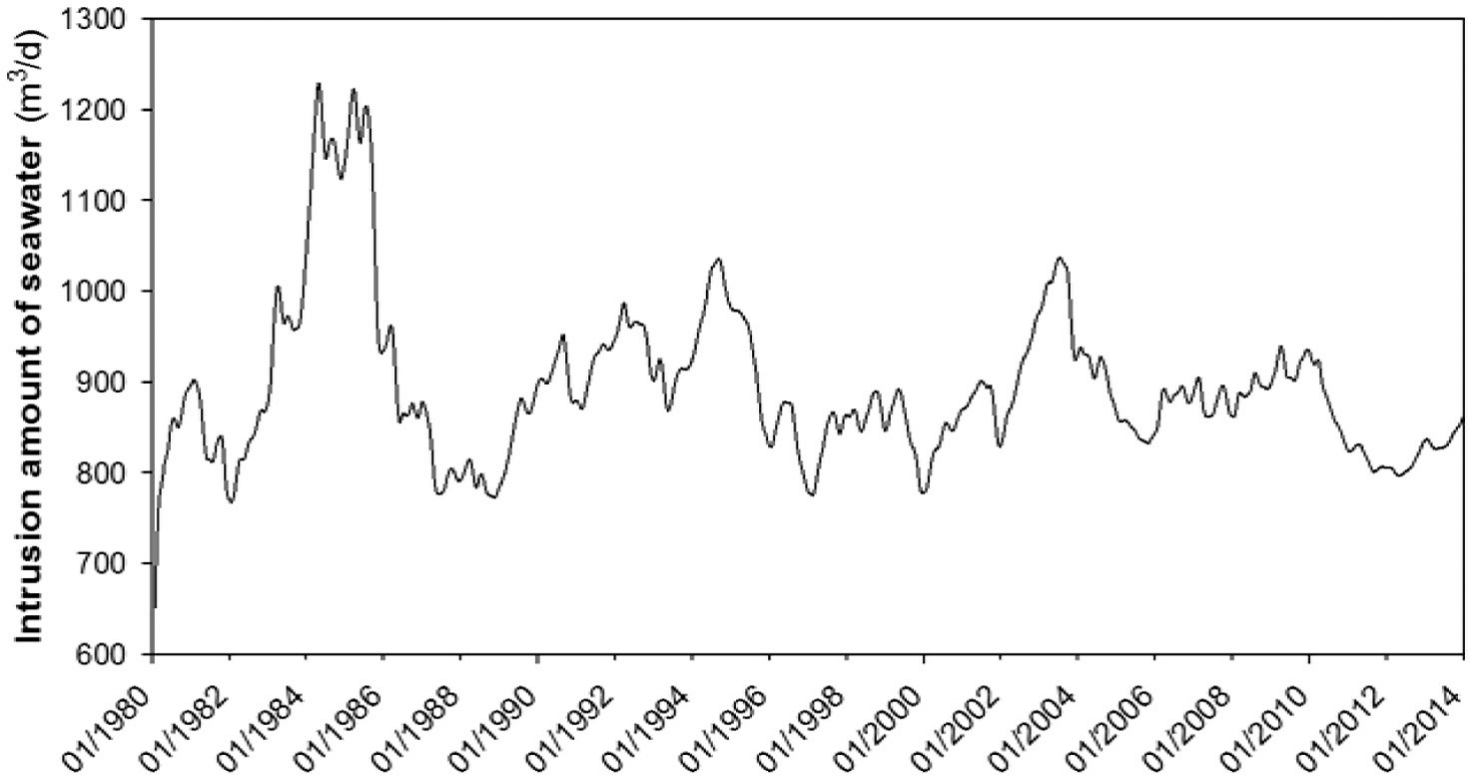
Seawater intrusion amount vs. time step (Transient Model).

Seawater intrusion is often associated with over pumping in coastal regions, resulting in overdraft conditions and creating an inland gradient of seawater [37]. Despite the fact that the total recharge amount matches the total groundwater withdrawal rate, in the dry season and high pumpage periods some portion of recharge to the aquifer comes from seawater of Atlantic Ocean due. The seawater intrusion to this karst aquifer versus time step hydro-diagram is shown in Figure 12. The largest discharge of groundwater (13,479 m^3^/d) from the karst aquifer to Atlantic Ocean occurred at February 1985. While during the same period, a total volume of 1230 m^3^/d seawater flowed into the public water supply well Boquillas (PS) in Manatí (see Figure 12), as a result of over pumping in this region. The facts that water budget is well balance and groundwater was overdraft in the Boquillas (PS) pumping area of Manti region indicate the seawater intrusion into this aquifer, which potentially leads to decline of groundwater quality by raising salinity to levels exceeding acceptable drinking and irrigation water standards and endangering future exploitation of coastal aquifers.

The intrusion of seawater into groundwater systems will also impact coastal ecosystems such as marshes by changing the elevation of the freshwater-saltwater interface [38]. Generally, pumping from public supply wells in coastal aquifers underlain by saltwater can lower the water table, decreasing the depth to the freshwater-saltwater interface beneath the pumping well. This increases the potential for saltwater intrusion, and potentially limits the amount of fresh water available from the well. Therefore, our study strongly suggested the control of pumping activities in the coastal region of this study area.

## 5. Conclusions

This study applied a three-dimensional equivalent porous media (EPM) approach to simulate regional groundwater flow in a highly permeable karstic aquifer and to provide data for planning water and management purposes. The groundwater system in the Manatí-Vega Baja area was simulated in three-dimensions in steady state and transient conditions using MODFLOW to investigate the aquifer potentiality with emphasis on groundwater flow direction, head distribution and mass balance. We conclude that the EPM approach was adequate in representing the hydrodynamics of the Manatí-Vega Baja karst aquifer despite the high potential of conduit dominated flow. The developed conceptual model can be applied further to predict the changes of groundwater levels and mass balance under different exploitation scenarios to predict future water demands in the study area. The results of water budget simulation reveals that the total outflow and inflow water to be equal in the aquifer and that the groundwater level of the aquifer tends to increase in future simulations. The simulated flow field showed that the regions around water supplying Boquillas pumping area is subject to seawater intrusion and decreased water quality due to salinization.

## Figures and Tables

**Table 1.** USGS monitoring well water table fluctuation range and total well depths.

No.	Well Name	WT Fluctuation Range (m)	Total Depths (m)
1	Cantito Laluisa Well Manatí PR	1.2 - 2.4	38.41
2	USGS 166 Observation Well Manatí PR	2.0 - 3.4	34.75
3	Coto Sur 5 Well Manatí PR	5.2 - 8.0	83.82
4	Rosario 2 Well Vega Baja PR	5.8 - 7.2	76.20

**Table 2.** Water budget of the aquifer for steady state condition (1995).

Flow Budget Term	Flow In (m^3^/d)	Flow Out (m^3^/d)
Constant Heads	128	−2629.74
Drains	0	−12,983.35
General Heads	939.72	−7785.47
Rivers	20,685.61	−12,508.15
Wells	5904.9	−79,726.56
Recharge	87,975.08	0
Total (Simulated)	115,633.31	−115,633.27
Total (Gregory 2001)	118,879.106	−118,903.572
Discrepancy (Simulated)	0.04%	
Discrepancy (Gregory 2001)	−24.47%	
